# Fluorination of Organic Spacer Impacts on the Structural and Optical Response of 2D Perovskites

**DOI:** 10.3389/fchem.2019.00946

**Published:** 2020-01-28

**Authors:** Inés García-Benito, Claudio Quarti, Valentin I. E. Queloz, Yvonne J. Hofstetter, David Becker-Koch, Pietro Caprioglio, Dieter Neher, Simonetta Orlandi, Marco Cavazzini, Gianluca Pozzi, Jacky Even, Mohammad Khaja Nazeeruddin, Yana Vaynzof, Giulia Grancini

**Affiliations:** ^1^Group for Molecular Engineering of Functional Materials, Institute of Chemical Sciences and Engineering, EPFL Valais Wallis, Sion, Switzerland; ^2^Laboratory for Chemistry of Novel Materials, Department of Chemistry, Université de Mons, Mons, Belgium; ^3^Univ Rennes, ENSCR, CNRS, ISCR (Institut des Sciences Chimiques de Rennes) - UMR 6226, Rennes, France; ^4^Integrated Centre for Applied Physics and Photonic Materials and Centre for Advancing Electronics Dresden (CFAED), Technical University of Dresden, Dresden, Germany; ^5^Institute of Physics and Astronomy, University of Potsdam, Potsdam, Germany; ^6^Young Investigator Group Perovskite Tandem Solar Cells, Helmholtz-Zentrum Berlin für Materialien und Energie GmbH, Berlin, Germany; ^7^CNR - Istituto di Scienze e Tecnologie Chimiche “G. Natta” (CNR-SCITEC), Milan, Italy; ^8^Univ Rennes, INSA Rennes, CNRS, Institut FOTON - UMR 6082, Rennes, France; ^9^Dipartimento di Chimica Fisica, University of Pavia, Pavia, Italy

**Keywords:** fluorinated organic spacer, 2D perovskites, phase transition, temperature dependence, excitonic materials

## Abstract

Low-dimensional hybrid perovskites have triggered significant research interest due to their intrinsically tunable optoelectronic properties and technologically relevant material stability. In particular, the role of the organic spacer on the inherent structural and optical features in two-dimensional (2D) perovskites is paramount for material optimization. To obtain a deeper understanding of the relationship between spacers and the corresponding 2D perovskite film properties, we explore the influence of the partial substitution of hydrogen atoms by fluorine in an alkylammonium organic cation, resulting in (Lc)_2_PbI_4_ and (Lf)_2_PbI_4_ 2D perovskites, respectively. Consequently, optical analysis reveals a clear 0.2 eV blue-shift in the excitonic position at room temperature. This result can be mainly attributed to a band gap opening, with negligible effects on the exciton binding energy. According to Density Functional Theory (DFT) calculations, the band gap increases due to a larger distortion of the structure that decreases the atomic overlap of the wavefunctions and correspondingly bandwidth of the valence and conduction bands. In addition, fluorination impacts the structural rigidity of the 2D perovskite, resulting in a stable structure at room temperature and the absence of phase transitions at a low temperature, in contrast to the widely reported polymorphism in some non-fluorinated materials that exhibit such a phase transition. This indicates that a small perturbation in the material structure can strongly influence the overall structural stability and related phase transition of 2D perovskites, making them more robust to any phase change. This work provides key information on how the fluorine content in organic spacer influence the structural distortion of 2D perovskites and their optical properties which possess remarkable importance for future optoelectronic applications, for instance in the field of light-emitting devices or sensors.

## Introduction

Hybrid organic–inorganic perovskites (HP) are currently one of the most attractive fields of research due to their large potential for photovoltaic applications. The performance of HP solar cells has enormously increased in the last decade (Correa-Baena et al., 2017), with power conversion efficiency of 25.2% (NREL solar cell efficiency chart., 2019), making this material one of the “hottest research topic” of our time, despite the issue related to device instability (Wang et al., 2019). More recently, a parent family, so called two dimensional (2D) HP, came into the limelight, largely because of their intriguing optical properties and higher environmental resistance, which offer new approaches to stabilizing perovskite-based photovoltaic devices (Misra et al., 2017). In addition, these low dimensional perovskites show unprecedented physical properties, making them a perfect test-bed samples for new fundamental chemical and physical understanding (Marongiu et al., 2019). It is worth to point out that vertical quantum and dielectric confinement effects in 2D perovskites enhance the single particle band gap by ~1 eV and the exciton binding energy by more than an order of magnitude (Straus and Kagan, 2018) with respect to the parented three dimensional (3D) perovskite from ~10 meV in 3D perovskites (Miyata et al., 2015; Yang et al., 2016) to > 400 meV in 2D perovskites with *n* =*1* (*n* = number of inorganic layers) (Keldysh, 1979; Kumagai and Takagahara, 1989; Hong et al., 1992; Ishihara et al., 1992; Tanaka and Kondo, 2003; Blancon et al., 2018; García-Benito et al., 2018). Thus, 2D perovskites present efficient narrow-band emission from excitonic resonance and bi-exciton emission, which provide them with an striking potential to be applied in optoelectronic applications, such as light-emitting devices (Mao et al., 2017; Chen et al., 2018). Moreover, 2D perovskites also exhibit anisotropic carrier transport which led to early field-effect transistor demonstrations (Senanayak et al., 2017; Zhang et al., 2019). The structure of these 2D perovskites with *n* = *1*, presents the general formula C_2_PbX_4_ where X^−^ is a halide anion and C^+^ refers to a large organic aromatic or aliphatic ammonium cation (Stoumpos et al., 2016). In this particular case, the inorganic frame has a unique configuration: it consists of one sheet of corner shared PbX_6_ octahedra, with thickness corresponding to just one PbX_6_, sandwiched within two layers of organic ammonium cations, acting as organic spacers. The incorporation of large cations relaxes dimensional constraints typical of traditional 3D perovksites, opening up a large choice on the organic moieties, ultimately enabling larger structural tunability. In the case of common aromatic or aliphatic ammonium cations, the 2D materials also show an intrinsic quantum-well electronic structure, where the frontier orbitals are in most cases fully confined within the semiconducting inorganic sheet and the insulating organic layer acts as electronic barrier (Traore et al., 2018). In this perspective, the electronic structure of the frontier orbitals is fully related to atomic contributions from the inorganic PbX_4_ frame, with hence no direct contribution on the electronic and optical response of the organic spacer. Notably, it has been widely reported how changing the halogen as well as the size and nature of the organic cations offer interesting features in 2D perovskites, such as superior hydrophobicity and high tunability of chemical composition and physical properties (Yu et al., 2007; Misra et al., 2017; Chen et al., 2018; Shi et al., 2018; Zheng et al., 2018). In particular, Du et al. explored the impact of organic and inorganic choice on the exciton properties. These authors studied a series of 2D perovskite crystals, based on different acene alkylamine cations and lead (II) halide (i.e., ${\text{PbX}}_{4}^{2-}$, *X* = Cl, Br, and I) frameworks. The results revealed that perovskite layer distortion (i.e., Pb–I–Pb bond angle between adjacent PbI_6_ octahedra) has a more global effect on the exciton properties than framework distortion (i.e., variation of I–Pb–I bond angles and discrepancy among Pb–I bond lengths within each PbI_6_ octahedron) (Du et al., 2017). Gan et al. studied the exciton recombination process as a function of the organic cation length. They provided physical understanding of the role of organic cation in the optical properties of 2D layered perovskites, particularly in the enhance of luminescence efficiency of such materials (Gan et al., 2017). Smith et al. demonstrated that incorporation of polarizable molecules in the organic layers, through intercalation or covalent attachment, is a viable strategy for tuning the exciton binding energy of 2D perovskites toward mimicking the reduced electronic confinement and isotropic light absorption of 3D perovskites (Smith et al., 2017). Kamminga et al. focused on additional lateral electronic confinement effects in systems with partial corner-sharing mode of octahedra, containing perovskite subnetworks (Kamminga et al., 2016) and recently, Van Gompel et al. demonstrated that it is possible to self-assemble organic charge-transfer complexes in the organic layer. Therefore, combinations of donors and acceptors could be targeted to obtain 2D perovskites where the organic and inorganic layer have synergistic properties (Van Gompel et al., 2019). Based on the influence of the organic spacer in 2D perovskites, different chemical composition has been explored as well (Wei et al., 2014; Chen et al., 2018). In particular, 2D perovskites featuring fluorinated alkyl or aryl ammonium cations have been previously proposed such as (FC_2_H_4_NH_3_)_2_PbCl_4_ (Lermer et al., 2016) and (*p-*FC_6_H_4_(CH_2_)_2_NH_3_)_2_PbX_4_ (*X* = Br^−^, I^−^ or Cl^−^) (Even et al., 2012; Wei et al., 2012, 2013). Crystals of new hybrid tin(II) iodide-based perovskites, involving 2,3,4,5,6-pentafluorophenethylammonium or phenethylammonium cation bilayers and intercalated aryl or perfluoroaryl molecules, were explored by Mitzi et al. (2002). In a recent work, we succeeded in incorporating heavily fluorinated organic cations into 2D structure, demonstrating the increased hydrophobicity and stability of the new lead-iodide 2D perovskites (García-Benito et al., 2018). The inclusion of fluorinated cations furnished the perovskite material with interesting structural/optical properties, inducing considerable strains in the PbI_4_ network, because of the bulkier fluorine atom, which in turn resulted in a change of the optical properties. However, a detailed understanding of fluorinated 2D perovskites, especially the control of their optical properties with temperature is still lacking. In this work, we explore the influence of fluorination of the organic cation in *n* = *1* 2D perovskite structure at room temperature (r.t.) and we investigate changes in the optical spectrum down to 80 K. To this purpose, we study two 2D perovskite structures in the form of C_2_PbI_4_, being C^+^ the well-known aliphatic cation C_9_H_19_${\text{NH}}_{3}^{+}$ (Dolzhenko et al., 1986; Ishihara et al., 1990; Billing and Lemmerer, 2008) here on named Lc, and its partially fluorinated analog C_9_H_6_F_13_${\text{NH}}_{3}^{+}$, here on named Lf, which we recently synthesized (García-Benito et al., 2018). In particular, temperature dependence ultraviolet–visible absorption spectroscopy (UV-Vis) and photoluminescence (PL) measurements were carried out to monitor the response of fluorinated and non-fluorinated 2D perovskites. Interestingly, phase transitions detected for the fully hydrogenated Lc compound (Billing and Lemmerer, 2008), which manifest with the appearance of multiple excitonic resonance at low temperature, disappear for the fluorinated Lf compound. The fluorination imparts more structural rigidity which also affects the optoelectronic properties. The Lc 2D perovskite shows a red-shifted excitonic emission and a doubled photoluminescence quantum yield. Additionally, it was found that the atomic substitution in the organics has an influence in the electronic density of states, which was monitored by ultraviolet photoelectron spectroscopy (UPS) and photothermal deflection spectroscopy (PDS). A decrease of 0.3 eV in the ionization potential (IP) was found as a result of incorporating fluorinated spacer in the 2D network.

## Results and Discussion

### Structural and Optical Characterization

**Figure 1:**
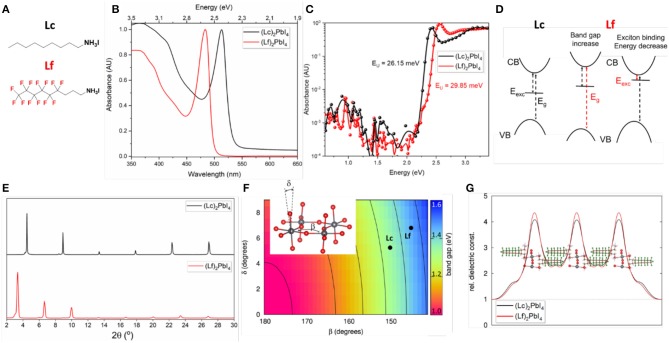
**(A)** Molecular structure of the organic spacers. **(B)** Absorbance and **(C)** PDS measurements of (Lc)_2_PbI_4_ and (Lf)_2_PbI_4_. **(D)** Schematic effect associated to the change of optical properties going from Lc to Lf, as related to band gap (E_g_) opening and decrease of the exciton binding energy. **(E)** XRD patters for (Lc)_2_PbI_4_ and (Lf)_2_PbI_4_ thin films. **(F)** Map of the band gap as function of the octahedral tilting associated to the angles β and δ (the definition of the angles is showed in the inset). **(G)** Dielectric profile of (Lc)_2_PbI_4_ and (Lf)_2_PbI_4_.

Low dimensional perovskites belong to a large family of structures which read as R_2_(A)_n−1_M_n_X_3n+1_, where R is the bulky organic molecule, A the small organic cation, M the divalent metal, X the halogen and *n* the number of inorganic layers (Mitzi, 2001). For *n* = *1*, one layer of corner-shared ${\text{MX}}_{6}^{4-}$ octahedral is sandwiched in between two arrays of large organic spacer. Figure 1A shows the molecular structure of the organic ammonium salts (Lc and Lf) investigated in this work. They both feature a nine-carbon alkyl chain, with the partial substitution of hydrogen for fluorine in Lf cation. The UV-Vis absorbance spectra at r.t of the corresponding (Lf)_2_PbI_4_ and (Lc)_2_PbI_4_ 2D perovskites in thin film are shown in Figure 1B. The strong excitonic peak dominates the spectra in both cases, a characteristic feature of 2D structures. Interestingly, despite the fact that the organic component does not contribute to the frontier orbitals of the 2D perovskite (Traore et al., 2018), we found a stark blue-shift of the excitonic resonance as a result of the presence of fluorine in the organic spacer. The lowest exciton band of Lc and Lf appear at 2.4 eV (513 nm) and 2.6 eV (483 nm), respectively, with a blue-shift of 0.2 eV. Further comparison with the well-studied 2D perovskite (BuA)_2_PbI_4_ (based on *n*-butylammonium cation, BuA) shows exactly the same exitonic peak position as (Lc)_2_PbI_4_ (see Figure S1). In parallel, also the continuum feature related to the onset of the band-to-band transitions, is blue shifted by compatible quantity (above 2.7 eV and 2.9 eV for the Lc and Lf, respectively). We note that this blue-shift is also present in photothermal deflection spectroscopy measurements (Figure 1C), which also allow us to quantify the energetic disorder in the samples by determining the Urbach Energy (E_U_). It is interesting that despite the similarities in the 2D structure of the films, Lf exhibits a larger E_U_ of 29.85 meV, as compared to only 26.15 eV for Lc. This suggests that the energetic disorder in Lf is higher, possibly due to larger distortion of the perovskite structure (see Figure 1D). It is also worth mentioning that form a microscopic point of view, different Urbach Energy can also partially derive from slight differences in film morphology and crystal quality of the two samples which are averaged out in the absorption measurements. Figure 1E shows the X-Ray Diffraction (XRD) patterns of the two films. The low angles diffraction signals are indicative of 2D perovskite *n* = *1* structures (Cao et al., 2015; Mao et al., 2016; Stoumpos et al., 2016). Specifically, the patterns are dominated by equally spaced (00l) reflections, peaking at 4.5° and 3.4° for (Lc)_2_PbI_4_, and (Lf)_2_PbI_4_, respectively. The reflections of the other (hkl) planes are strongly suppressed, confirming the remarkably preferred orientation of the inorganic planes, aligning parallel to the substrate (Mao et al., 2016; Stoumpos et al., 2016). The distance between the inorganic planes results in spacing of 26.75 and 20.07 Å for (Lf)_2_PbI_4_ and (Lc)_2_PbI_4_, respectively. The XRD pattern of (BuA)_2_PbI_4_ film is used for comparison (Figure S2).

These results raise an important question: what is the origin of the shift in the absorbance spectra, which induces a 0.2 eV blue-shift of the excitonic resonance. Conceptually, such change in the optical properties can be explained either as an opening of the electronic band gap upon introduction of Lf, or as a decrease of the exciton binding energy, or a combination of the two effects (see Figure 1D). The determination of the band gap from the UV-vis spectrum can be challenging in the case of excitonic materials, as the ones investigated here. In fact, the exact rise of the band-to-band transition is hidden by the excitonic resonance. Tentative estimation of the band gap via Tauc plot is reported in Figure S3, which suggests a blue-shift of 0.1 eV going from Lc to Lf. To support this analysis, we performed periodic Density Functional Theory (DFT) calculations, which directly evaluate the single particle band gap, in absence of excitonic effects. In the present case, the calculation is complicated by the lack of structural information for the Lf compound. In fact, while structural models based on XRD measurements are fully available from the literature for the Lc compound (Billing and Lemmerer, 2008), no structural models we available for the Lf one, with the only structural information available represented by the reported increase in interlayer distance illustrated in Figure 1E. We hence started from the orthorhombic structure reported for the Lc compound (Billing and Lemmerer, 2008) and we provided reference model for the Lf compound, by substituting the hydrogen with fluorine in the cation and increasing the interlayer distance, consistently with the datum in Figure 1E. Then, for both Lc and Lf, we performed structural relaxation of all the atomic parameters, keeping the lattice and space group fixed, so to obtain the equilibrium structure of the two compounds at zero K temperature. To accurately describe van der Waals interactions, which are expected to play a role on intermolecular interactions between organic chains, we used VDW-DF2 functional, which has been explicitly designed for this purpose (Lee et al., 2010). We then computed the single particle band gaps using the PBE exchange-correlation functional, including Spin-Orbit Coupling, which is known to largely affect the electronic properties of lead-based HP (Even et al., 2013), but without affecting the optical anisotropy of 2D perovskites (Even et al., 2012). For Lc and Lf, we found band gap, respectively, of 1.34 and 1.46 eV. The computed single particle band gaps are severely underestimated with respect to the experimental data, because of lack of accurate treatment of electronic exchange and correlation (Quarti et al., 2018), but on the other hand, it provides a blue-shift of the band gap of 0.12 eV going from Lc to Lf, which goes in the same direction of the Tauc plot analysis. In turn, this band gap opening upon cation substitution is very well-rationalized in terms of increased structural distortions in the inorganic plane, in agreement with the finding of Knutson et al. (2005). The tilting in the octahedra is conveniently described in terms of the angles β and δ as showed in the inset of Figure 1F. In the figure, the band gap of a purely structural model of 2D perovskite is mapped as function of the values of these two angles at the same level of theory (Pedesseau et al., 2016). As shown, the more the structure distorts (β and δ deviating from 180° and 0°, respectively), the more the band gap increases, because of the reduced atomic wavefunction overlap, and consequent decrease of band valence and conduction bandwidth (Umebayashi et al., 2003; Quarti et al., 2018; Traore et al., 2018). Nicely, the band gap computed for the structure of Lc and Lf well-fit in the 2D map in Figure 1F. As final remark, the lack of structural information for Lf and the use of in-plane lattice parameters from Lc can clearly influence the results of the present analysis. To check this possibility, we further relaxed both the atomic positions and the lattice parameters. In the present case, the obtained band gaps, 1.29 and 1.43 eV for Lc and Lf, respectively, are slightly different than the results obtained by fixing the lattice, but the blue-shift from the former to the latter compound is confirmed, hence reinforcing the present analysis. This structural effect is hence consistent with the band gap opening mechanism reported in Figure 1D. However, a possible effect of the organic cation on the exciton binding energy remains to be checked. Quantitative estimation of the exciton binding energy is on the other way a very hard task, as the inclusion of the electron-hole interaction requires the use of complex approaches, based on the Time-Dependent (TD)DFT method (Quarti et al., 2018) or on the solution of BSE performed on converged single particle electronic structure, including spin-orbit coupling (Giorgi et al., 2018). On the other hand, the computational cost associated to these calculations is very large and they are not yet able to describe the well-known deviations from 2D Rydberg series, making these methods unaffordable for the compounds investigated here and the requested level of accuracy. Alternatively, one can address changes in exciton binding energy by estimating the change in the dielectric inhomogeneity along the stacking axis, which enhance the screening between the photo-excited electron-hole pair by the remaining electrons (Katan et al., 2019). It is in fact reasonable to expect some change in the dielectric response, as results of the introduction of electron rich fluorine atoms. To this aim, we adopted the formalism developed in Even et al. (2014) and Sapori et al. (2016) which consists in performing DFT calculations in presence of external fields and to determine the profile of the dielectric response from the electron density re-organization. More importantly, by determining the dielectric profile along the normal to the inorganic layer, one is able to distinguish between the dielectric response of the inorganic and of the organic components. The dielectric profile computed for both Lc and Lf following this approach is shown in Figure 1G, and indicates that only very limited changes in the dielectric response are found upon introduction of fluorine atoms. In comparison, changes of the dielectric constant of a factor of 3 where reported upon intercalation of molecular I_2_ into 2D perovskites, with corresponding sizable change in the exciton binding energy (Smith et al., 2017). We conclude that the blue-shift observed in Figure 1B can be mainly attributed to a band gap opening, with negligible effects on the exciton binding energy.

### Temperature Dependence Optical Properties

**Figure 2:**
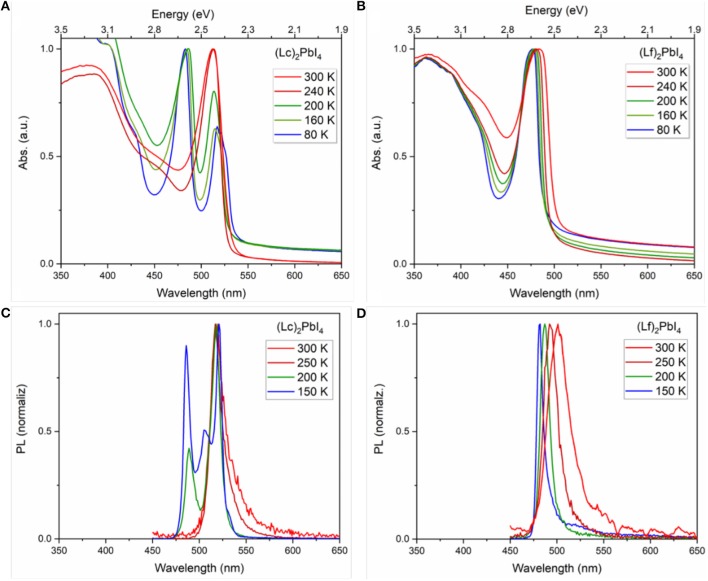
Temperature dependence in thin film: Absorbance for **(A)** (Lc)_2_PbI_4_ and **(B)** (Lf)_2_PbI_4_; and photoluminescence (λ_excitation_ = 367 nm) for **(C)** (Lc)_2_PbI_4_ and **(D)** (Lf)_2_PbI_4_.

Interesting differences can be observed in the optical response as a function of temperature, following the partial fluorination of the organic spacer. UV-Vis and PL measurements were carried out going from room temperature down to 80 K (see experimental methods for details). The results for (Lc)_2_PbI_4_ and (Lf)_2_PbI_4_ thin films are collected in Figure 2. The larger population of phonons found with increasing the temperature results in more frequent electron-phonon scattering events, which in turn determines the larger bandwidth of the peak observed at r.t., compared to low temperature measurements. Remarkably, in the case of fully-hydrogenated cation-based material, (Lc)_2_PbI_4_, a second peak at low wavelength (483 nm) appears between 240 and 200 K along with a decrease in the intensity of the main peak at 2.4 eV (517 nm) found at room temperature, indicating the formation of a second phase as a result of an alteration in the crystalline structure. Polymorphism in this class of materials is in fact quite common and widely reported. In particular, D. G. Billlings and A. Lehmerer reported a phase transition for Lc (Billing and Lemmerer, 2007, 2008; Lemmerer and Billing, 2012), at 240.9 K, from an orthorhombic to a monoclinic phase, hence in reasonable agreement with our optical data. The appearance of a second peak at higher energy is also observed in the case of shorter fully-hydrogenated cations such as butylammonium in the form of *n* = *1* 2D perovskite, (BuA)_2_PbI_4_ (see Figure S4), with similar phase transition observed in the literature (Billing and Lemmerer, 2007). The 28 nm difference in the peak positions (513–485 nm) is exactly the same for both fully-hydrogenated cations (BuA and Lc). Nevertheless, it is not to exclude a role of the morphology of the sample (thin-film) on the thermodynamics of the phase transition, which can therefore happen at different temperatures or that can result in the co-existence of two segregated phases. In contrast, the fluorinated Lf does not show any additional blue-shifted peak, which can indicate, a different structural arrangement of the material within the temperature range investigated. This indicates that the organic cation does play an ultimate role on the structural and optical response of layered 2D *n* = *1* HP. Interestingly one may observe that (Lc)_2_PbI_4_, (Lf)_2_PbI_4_ and (BuA)_2_PbI_4_ thin films exhibit very similar optical band gaps at low temperature (Figure 2, Figures S4, S5), thereby suggesting that the distortions of the (Lf)_2_PbI_4_ perovskite network are similar to the ones of (Lc)_2_PbI_4_ and (BuA)_2_PbI_4_ below the phase transition (vide infra). Previous literature about dielectric confinement effect of excitons in PbI_4_-based layered semiconductors corroborate the fact that the perovskite undergoes a phase transition upon cooling as a consequence of tuning the length and dielectric constant, for some organic cations (Ishihara et al., 1990; Barman et al., 2003; Naik and Vasudevan, 2010; Straus and Kagan, 2018). These phase transitions can drive changes in band structure that complicate in some cases the evaluation of temperature-dependent optical properties (Even et al., 2015). Surprisingly, a phase transition is absent for some non-fluorinated 2D perovskites such as (C_6_H_13_NH_3_)_2_PbI_4_ (Ishihara et al., 1990) and (PEA)_2_PbI_4_ (Hong et al., 1992), demonstrating the important influence of the cation on the thermodynamic properties of 2D HP. More recently, N. Kitazawa et al. have indeed systematically demonstrated that very subtle changes for alkyl-based cations in the barrier, may trigger of not abrupt low temperature structural phase transitions including hysteresis (Kitazawa et al., 2011). In addition, Yaffe et al. reported that interactions between 2D perovskites and the environment also affect phase transitions. As an example, they claim that the interaction of an exfoliated sheet with a silica substrate eliminates the freezing/melting phase transition in (BuA)_2_PbI_4_ (Yaffe et al., 2015), and this distinction must be taken into account when comparing the temperature-dependent optical properties of exfoliated sheets and larger single crystals.

**Figure 3:**
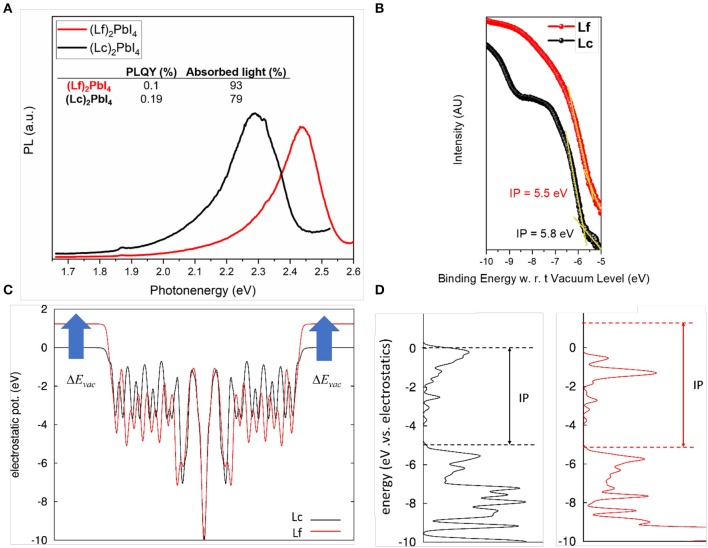
**(A)** Relative PLQY at 300 K and 1 sun for (Lc)_2_PbI_4_ and (Lf)_2_PbI_4_. **(B)** UPS measurements. The intensity scale is linear. The spectra were normalized to the same height for visual clarity. **(C,D)** Results of DFT calculations on slab models. **(C)** Electrostatic potential along the normal to the slab. **(D)** corresponding density of states and ionization potential.

We extended the comparison of the optical properties, by monitoring the emission properties of the fluorinated and non-fluorinated 2D perovskites from r.t. down to 150 K (Figures 2C,D). The emission spectra of (BuA)_2_PbI_4_ in temperature for comparison, can be found in Figure S5. The results confirm the trend observed in absorption. At room temperature both 2D perovskites present a single emission peak located at 519 nm and 501 nm for (Lc)_2_PbI_4_ and (Lf)_2_PbI_4_, respectively. However, the emission response at low temperature presents a second peak at lower wavelength (486 nm, 2.6 eV) for the non-fluorinated cation (Lc) in the range of 250–200 K. In contrast, the emission properties in the case of the fluorinated perovskite (Lf)_2_PbI_4_, is remarkable different. Only one peak is observed in all range of temperature studied, that indicates the absence of phase transition as a response to the reduction in temperature. Also, the maximum peak in the emission is getting narrower and blue-shifted when the temperature decreases (going from 501 to 482 nm). In both system, notably, no broadband emission is observed at low temperature. In addition, PL decays were monitored, and no appreciable differences were found in the dynamic response of these materials (see Figure S6). In contrast, a notable difference in the photoluminescence quantum yield (PLQY) is observed comparing fluorinated and non-fluorinated 2D perovskite structures. The obtained values fixing the light intensity at 1 sun, are promising and in line to what previously reported 2D perovskites (Cortecchia et al., 2017) being 0.1 % for (Lf)_2_PbI_4_ and the double, 0.2 % for (Lc)_2_PbI_4_ (see Figure 3A). As the PLQY is defined as the ratio of the emitted photon flux to the absorbed photon flux, it can be concluded that the 2D perovskite based on non-fluorinated organic spacer presents a more efficient radiative recombination of charges than the fluorinated one. This difference might be related to different film crystalline quality, ultimately associated to the different trap density in the two materials. This observation is in line with the absolute PL intensity behavior with lowering the temperature (see Figure S9). At low T the non-radiative processes and the trapping of charges are slowed down leading to an increase in the radiative efficiency. For the fluorinated sample, the PL peak increases 23-folds from high to low T, while for the non-fluorinated cation the increase is 18 times, indicative for a lower trap density for the latest. This finding highlights the importance of structural engineering, in particular the election of the organic spacer, to control the photoluminescent properties of this class of perovskite.

### Electronic Structure of 2D Perovskites

The energetics of the electrons in the valence and conduction bands represent another key parameter for the technologic exploitation of 2D HP. To have access to the density of occupied electronic states, including the energetics of the valence band maximum (hence the ionization potential, IP), as referred to the energy in the vacuum we performed ultraviolet-photoelectron spectroscopy measurements. The results of UPS measurements for Lc and Lf perovskites are shown in Figure 3B. The valence band (VB) position are extracted using a linear extrapolation of the leading edges in the UPS spectra in agreements with previous works (Gao, 2010; Irfan et al., 2010; Olthof and Meerholz, 2017; Fassl et al., 2018). The measured IP are 5.8 and 5.5 eV for Lc and Lf, respectively and are comparable to the reported IP of CH_3_NH_3_PbI_3_ (Schulz et al., 2014; Fassl et al., 2018). Notably, a change in IP (0.3 eV) was found in presence of the fluorinated Lf spacer. This result is in part surprising, as the inclusion of strongly electronegative atoms in Lf at opposite position to the positive ${\text{NH}}_{3}^{+}$ group is expected to provide additional negative contribution to the molecular dipole (see also Figures S7, S8). Quantum chemical calculations indeed provide a dipole moment of 23.07 Debye and 32.76 Debye, respectively for Lc and Lf, with the positive side on the ${\text{NH}}_{3}^{+}$ group. This increase in the dipole moment in turn should result in the up-shift of the electrostatic potential in the vacuum (*E*_*vac*_), as expressed by the Helmholz relation (Cahen and Kahn, 2003),

(1) $$\begin{array}{l} \Delta E_{vac}=-\frac{\mu_{z}}{\varepsilon_{0}A} \end{array}$$

(where **μ**_z_ is the electric dipole normal to the surface, A is the surface and ε_0_ is the dielectric constant) and consequently in the increased of the work function and of the Ionization potential. Periodic DFT calculations performed on slab models further confirm this picture. In Figure 3C, we report the electrostatic potential averaged along the normal of the slab, which clearly highlights the expected up-shift in the vacuum potential, from Equation (1), while in Figure 3D, we report the corresponding electron Density of States (DOS), showing the increase in the IP. We can speculate that this apparent contrast between theory and experiment could be related to non-idealities, as point defects (vacancies or interstitial of cations and anions) or different conformation of the spacers at the surface, which, considering the soft nature of the material and the solution technique employed for its fabrication, are not unlikely. It is also worth to point out that the determination of the IP of hybrid perovskites has always revealed challenging, highlighting a strong sensitivity of this quantity to the substrate (Olthof and Meerholz, 2017), surface defects (Haruyama et al., 2014), and termination (Quarti et al., 2017). Specifically, literature data shows dispersion for the UPS measured IP of the prototypical 3D CH_3_NH_3_PbI_3_ halide perovskite for more than 1 eV (Schnier et al., 2017), which indeed evidences a great sensitivity of the electronic properties of these materials, with respect to the detailed surface structure and synthesis conditions. In this sense, further characterization activity, properly supported by theoretical models, are thus required in order to clarify the mechanisms affecting the energetics of frontier orbitals in hybrid halide perovskites.

## Conclusions

In summary, we explored the influence of single atomic substitutions in the organic cation on structural, electronic and optical properties of 2D perovskites. In particular, hydrogen was partially replaced by fluorine atoms in the spacers allowing the comparison between fluorinated and non-fluorinated 2D perovskites, (Lf)_2_PbI_4_ and (Lc)_2_PbI_4_, respectively. As a result, a 0.2 eV blue-shift was observed in the excitonic position at r.t. that can be mainly attributed to a band gap opening, with negligible effects on the exciton binding energy. According to DFT calculation, the more the structure distorts, the more the band gap increases, because of the decreased atomic wavefunction overlap, and consequent decrease of band valence and conduction bandwidth. Interestingly, the atomic substitution in the organic moieties results in an absence of phase transitions at low temperature in the fluorinated perovskite, in contrast to widely reported polymorphism in some non-fluorinated materials leading to a phase transition. We can conclude that a small perturbation on the material structure strongly affects also the overall structural stability and related phase transition of the materials, making it more robust to any phase change. In addition, a moderate increase in the PLQY is observed comparing fluorinated and non-fluorinated 2D perovskite structures. The obtained values are promising comparing to previously reported 2D perovskites being 0.1% for (Lf)_2_PbI_4_ and the double, 0.2% for (Lc)_2_PbI_4_.

## Experimental Methods

### Synthesis of Ammonium Cations

4,4,5,5,6,6,7,7,8,8,9,9,9-Tridecafluorononylamine hydroiodide (Lf) was prepared as previously described (García-Benito et al., 2018). Nonylamine hydroiodide (Lc) was prepared by reaction of nonylamine (1 mL, 5.45 mmol) with 57% aqueous HI (0.85 mL, 5.6 mmol) in MeOH (6 mL). After 2 h stirring at rt, the solvent was removed at reduced pressure. The solid material was triturated with Et_2_O and Petroleum Ether, filtered and dried under reduced pressure to afford 1.44 g (97% yield) of the ammonium salt.

^1^H NMR (400 MHz, CDCl_3_) δ 7.56 (s br, 3H), 3.13 (m, 2H), 1.86 (m, 2H), 1.55 – 1.11 (m, 12H), 0.87 (t, *J* = 6.7 Hz, 3H). ^13^C NMR (101 MHz, CDCl_3_) δ 40.58, 31.84, 29.38, 29.22, 28.99, 27.38, 26.67, 22.69, 14.13.

### Preparation of Thin Film

The fabrication of 2D perovskite thin films followed a similar procedure. First, 1.2 M of PbI_2_ was dissolved in DMSO at 60°C. Then, the solution was cooling down and 2.4 M of the organic cation was added to the solution. The solution was then deposited onto the substrate via a consecutive two-step spin-coating process at 1,000 rpm for 10 s and 5,000 rpm for 30 s. During the second step, 100 μL of chlorobenzene was deposited. No antisolvent was used to for the 2D perovskite based on Lc cation. The resulting films were then annealed at 100°C for 15 min.

### X-Ray Diffraction (XRD) Characterization

XRD patterns were recorded by X-ray diffractometer (Bruker D8) with Cu kα radiation.

### Absorption and Photoluminescence

UV-Vis steady-state absorption spectra were acquired with a Perkins Elmer lambda 950 s UV/Vis spectrophotometer using an integrating sphere.

### Steady State and Time Resolved Photoluminescence

Steady state and time resolved photoluminescence measurement where carried out on Horiba a Fluorolog-3, with a PMT as detector. The excitation source for the TCSPC is a Horiba nanoLED370 with an excitation wavelength of 369 nm, a pulse duration of 1.3 ns and a repetition rate of 100 KHz. PL at different intensity was performed using an Expla PL 2230 laser coupled to a PG400 OPG. The spectra are recorded with a Horiba synapse CCD detector coupled to a monochromator. Steady state and time resolved photoluminescence measurement where carried out on Horiba a Fluorolog-3, with a PMT as detector. The excitation source for the TCSPC is a Horiba nanoLED-370 with an excitation wavelength of 369 nm, a pulse duration of 1.3 ns and a repetition rate of 1 MHz.

### Ultraviolet Photoemission Spectroscopy (UPS)

Films of 2D perovskites on FTO/glass were transferred into an ultrahigh vacuum (UHV) chamber of the PES system (Thermo Scientific ESCALAB 250Xi) for measurements. UPS measurements were carried out using a double-differentially pumped He discharge lamp (hν = 21.22 eV) with a pass energy of 2 eV and a bias at −5 V.

### Photothermal Deflection Spectroscopy (PDS)

Perovskite layers for PDS characterization were prepared on spectrosil in an identical way to those on glass/FTO. The samples were mounted in a sample holder filled with Fluorinert FC-770 (IoLiTec) in a nitrogen filled glovebox. A 150 W xenon short-arc lamp (Ushio) provides light for a monochromator (Cornerstone 260 Oriel, FWHM 16 nm) to achieve a chopped, tunable, monochromatic pump beam. The heat caused through absorption of the pump light in the perovskite films changes the refractive index of the Fluorinert. This change is detected by deflecting a probe He–Ne-laser (REO) whose displacement in turn is measured by a position-sensitive-detector (Thorlabs, PDP90A). The magnitude of the deflection is determined by a lock-in amplifier (Amatec SR 7230) and directly correlated to the absorption of the film. To estimate the Urbach energies a python leastsquare routine is used to fit an Urbach tail to the measured absorption spectra in the range of the absorption edge.

### Absolute Photoluminescence

Excitation for the PL measurements was performed with a 445 nm CW laser (Insaneware) through an optical fiber into an integrating sphere. The intensity of the laser was adjusted to a 1 sun equivalent intensity by illuminating a 1 cm^2^-size perovskite solar cell under short-circuit and matching the current density to the *J*_*SC*_ under the sun simulator (22.0 mA/cm^2^ at 100 mWcm^−2^, or 1.375 × 10^21^ photons m^−2^s^−1^). A second optical fiber was used from the output of the integrating sphere to an Andor SR393i-B spectrometer equipped with a silicon CCD camera (DU420A-BR-DD, iDus). The system was calibrated by using a calibrated halogen lamp with specified spectral irradiance, which was shone into to integrating sphere. A spectral correction factor was established to match the spectral output of the detector to the calibrated spectral irradiance of the lamp. The spectral photon density was obtained from the corrected detector signal (spectral irradiance) by division through the photon energy (*hf*), and the photon numbers of the excitation and emission obtained from numerical integration using Matlab. In a last step, three fluorescent test samples with high specified PLQY (~70%) supplied from Hamamatsu Photonics where measured where the specified value could be accurately reproduced within a small relative error of <5%.

### Computational Methods

Periodic and molecular electronic structure calculations are performed in the frame of the Density Functional Theory (DFT). Crystal structure relaxation (including both fixed and variable lattice calculations) and band gap estimates are performed using the pseudopotential/plane-wave approach, as implemented in the PWSCF code (Giannozzi et al., 2017). For these calculations, we considered a cutoff of 25 Ry and 200 Ry for the plane-wave expansion of the electronic wavefunction and density, along with ultrasoft pseudopotentials, and a 4 × 4 × 1 mesh of the first Brillouin zone, in the Monkhorst-Pack scheme (the smallest sampling factor is associated to the stacking plane direction, in relation to the corresponding larger parameter in the direct lattice). The exchange-correlation potential employed is cited throughout the text. The dielectric response of the Lc and Lf materials are calculated with the Siesta package (Soler et al., 2001), using the same sampling of the first Brillouin zone, along with double-split quality basis set and PBE functional for the description of the exchange and correlation. We verified the convergence of long-range slab to slab interactions, as reported in the Supporting Information. Finally, we computed molecular properties, i.e., molecular dipole moment, of Lc and Lf using Gaussian16 (Frisch et al., 2016), doting double split 6–31G(d,p) basis set along with PBE exchange correlation functional, for a matter of consistency with the previous calculations. Notably, test calculations with hybrid B3LYP functional provided molecular dipoles in close agreement with those from PBE.

## Data Availability Statement

All datasets generated for this study are included in the article/Supplementary Material.

## Author Contributions

IG-B, GG, and YV planned the experiments, prepared the 2D perovskites, carried out the absorption in temperature, the XRD measurements and analysis. VQ performed the PL temperature measurements. CQ and JE carried out the theoretical analysis. PC carried out the PLQY measurements. SO, MC, and GP synthesized and provided the organic cations. GG guided and supervised the project. IG-B and CQ wrote the manuscript which has been edited by all co-authors. All the authors participated in results discussion and analysis.

### Conflict of Interest

The authors declare that the research was conducted in the absence of any commercial or financial relationships that could be construed as a potential conflict of interest. The reviewer DC declared a past co-authorship with one of the authors CQ to the handling editor.
