# Supplementary material for: Fluorination of Organic Spacer Impacts on the Structural and Optical Response of 2D Perovskites
Source: Front Chem. 2020 Jan 28;7:946. doi: 10.3389/fchem.2019.00946 (PMC6999157; doi:10.3389/fchem.2019.00946)
Supplement: Supplementary file 1 [file Table_1.docx]

Supplementary Material

Fluorination of organic spacer impacts on the structural and optical response of 2D perovskites

Inés García-Benito,^1^ Claudio Quarti,^2,3*^ Valentin I. E. Queloz,^1^ Yvonne J. Hofstetter,^4^ David Becker-Koch,^4^ Pietro Caprioglio,^5,6^ Dieter Neher,^5^ Simonetta Orlandi,^7^ Marco Cavazzini,^7^ Gianluca Pozzi,^7^Jacky Even,^2^ Mohammad Khaja Nazeeruddin,^1^ Yana Vaynzof,^4^ Giulia Grancini^1,8*^

^1^Group for Molecular Engineering of Functional Materials, Institute of Chemical Sciences and Engineering, EPFL Valais Wallis, Sion, Switzerland

^2^Univ Rennes, INSA Rennes, CNRS, Institut FOTON - UMR 6082, Rennes, France.

^3^Laboratory for Chemistry of Novel Materials, Department of Chemistry, Université de Mons, Mons, Belgium

^4^Integrated Centre for Applied Physics and Photonic Materials and Centre for Advancing Electronics Dresden (cfaed), Technical University of Dresden, Dresden, Germany

^5^University of Potsdam, Institut für Physik und Astronomie, Potsdam, Germany

^6^ Young Investigator Group Perovskite Tandem Solar Cells, Helmholtz-Zentrum Berlin für Materialien und Energie GmbH, 12489 Berlin, Germany

^7^CNR - Istituto di Scienze e Tecnologie Chimiche “G. Natta” (CNR-SCITEC), Via Golgi 19, 20133 Milano, Italy

^8^Dipartimento Di Chimica Fisica, University of Pavia, Pavia, Italy

*** Correspondence:** claudio.quarti@umons.ac.be; giulia.grancini@unipv.it

Figure S1 shows the absorbance at room temperature of the investigated 2D perovskites (Lc)_2_PbI_4_ and (Lf)_2_PbI_4_) in thin film compared to (BuA)_2_PbI_4_.





Figure S1. Absorbance of (BuA)_2_PbI_4_, (Lc)_2_PbI_4_ and (Lf)_2_PbI_4_.in thin film at r.t.

Figure S2 shows the XRD of the investigated 2D perovskites ((Lc)_2_PbI_4_ and (Lf)_2_PbI_4_) in thin film compared to (BuA)_2_PbI_4_.





Figure S2. XRD pattern for thin films of (BuA)_2_PbI_4_, (Lc)_2_PbI_4_ and (Lf)_2_PbI_4_.

Tentative estimation of the band gap via Tauc plot is reported in Figure S3 for (Lc)_2_PbI_4_, (Lf)_2_PbI_4_ and (BuA)_2_PbI_4_ in thin film.

*
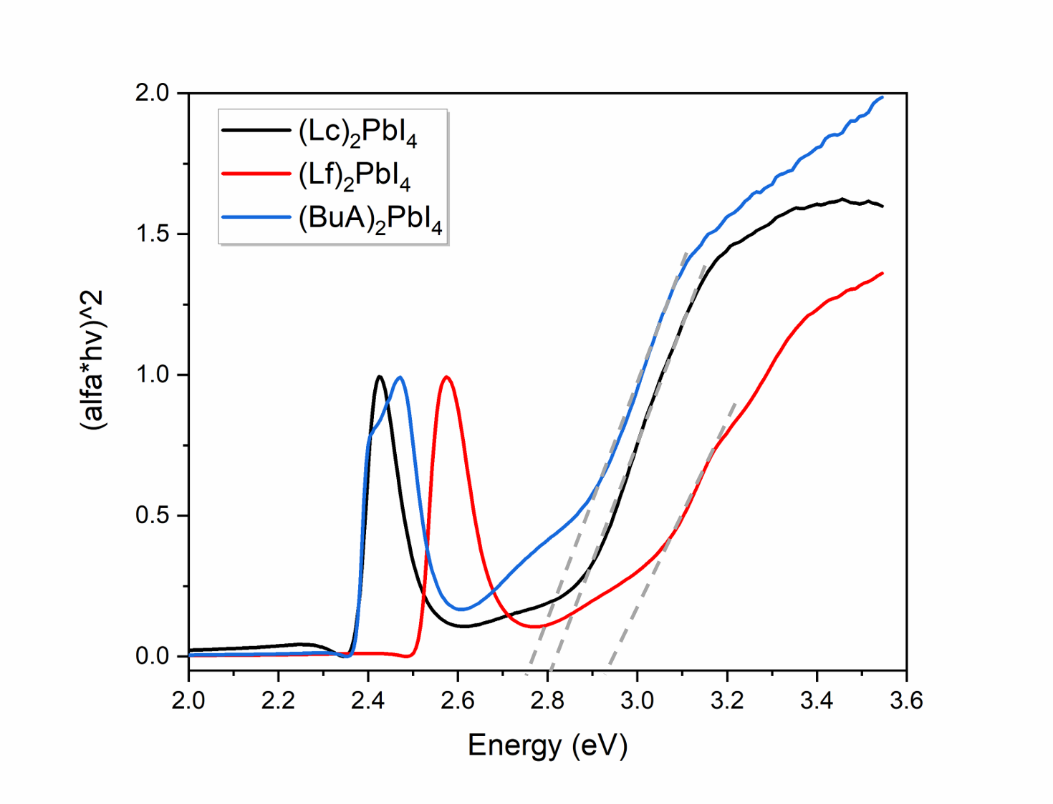
*

Figure S3. Tauc Plot for (BuA)_2_PbI_4_, (Lc)_2_PbI_4_ and (Lf)_2_PbI_4_.

Temperature dependence absorbance is illustrated in Figure S4 for (BuA)_2_PbI_4_ in thin film.





Figure S4. Temperature dependence absorbance for (BuA)_2_PbI_4_ in thin film.

Temperature dependence emission is illustrated in Figure S5 for (BuA)_2_PbI_4_ in thin film.





Figure S5. Temperature dependence photoluminescence for (BuA)_2_PbI_4_ in thin film.

PL decay is illustrated in Figure S6 for three investigated 2D perovskites in thin film.





Figure S6. Normalized PL decay of (BuA)_2_PbI_4_, (L9c)_2_PbI_4_ and (L)_2_PbI_4_ in thin ﬁlms at room temperature. (λ_excitation_ = 370 nm)


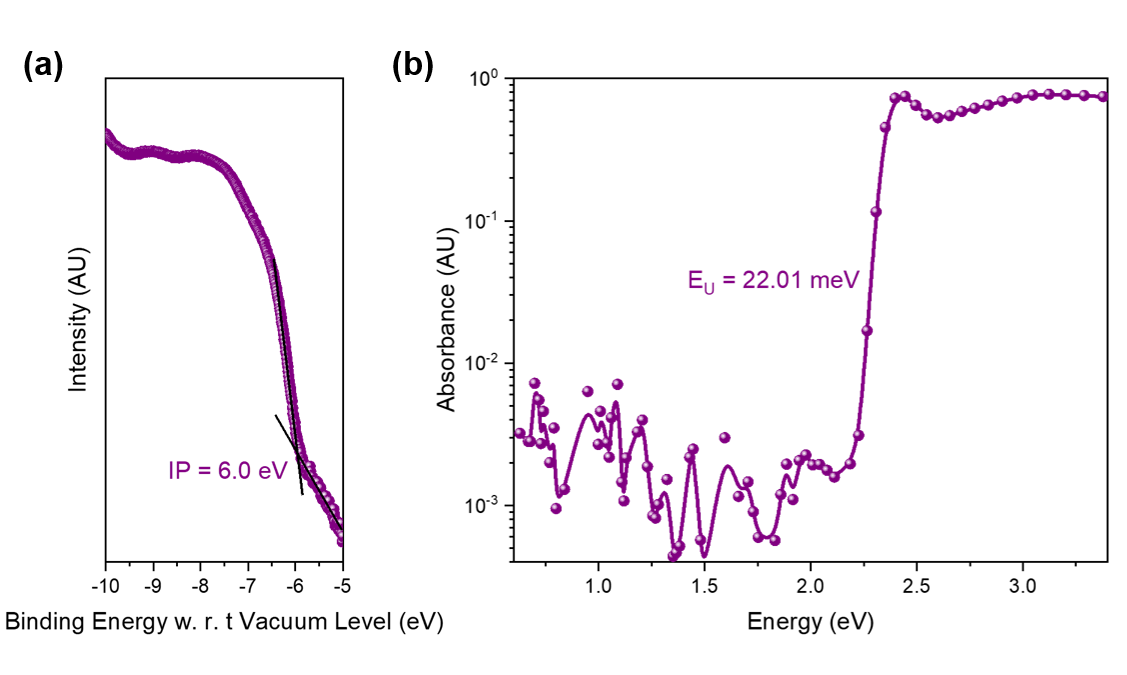


Figure S7. a) UPS and b) PDS spectra for (BuA)_2_PbI_4_ in thin film.


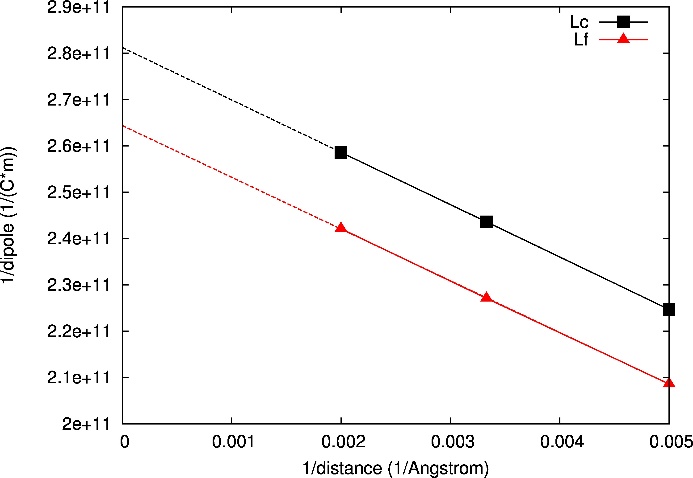


Figure S8. Convergence of long-range induced dipole-induced dipole interaction in the calculation of the dielectric response of slabs of Lc and Lf materials.


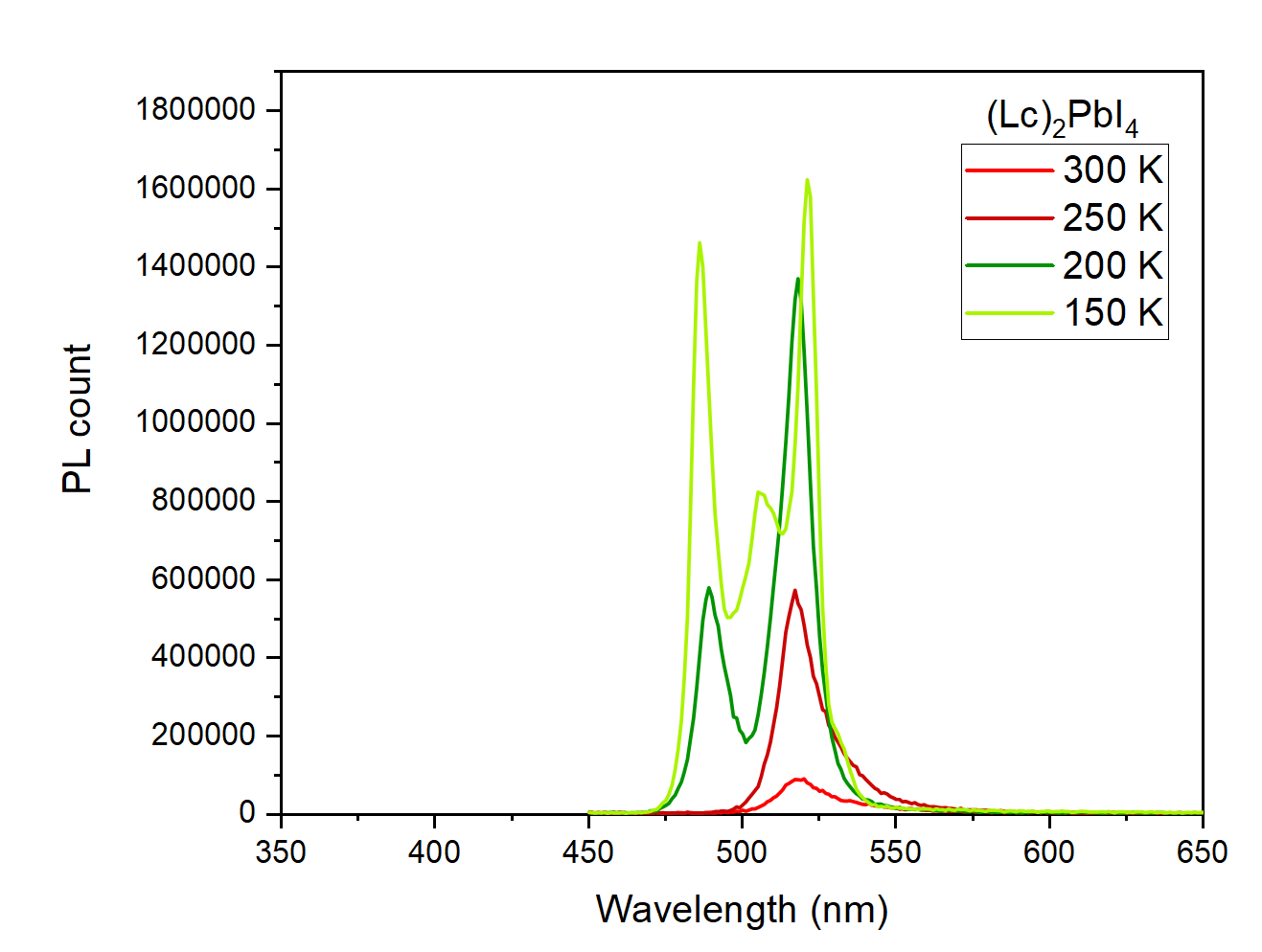

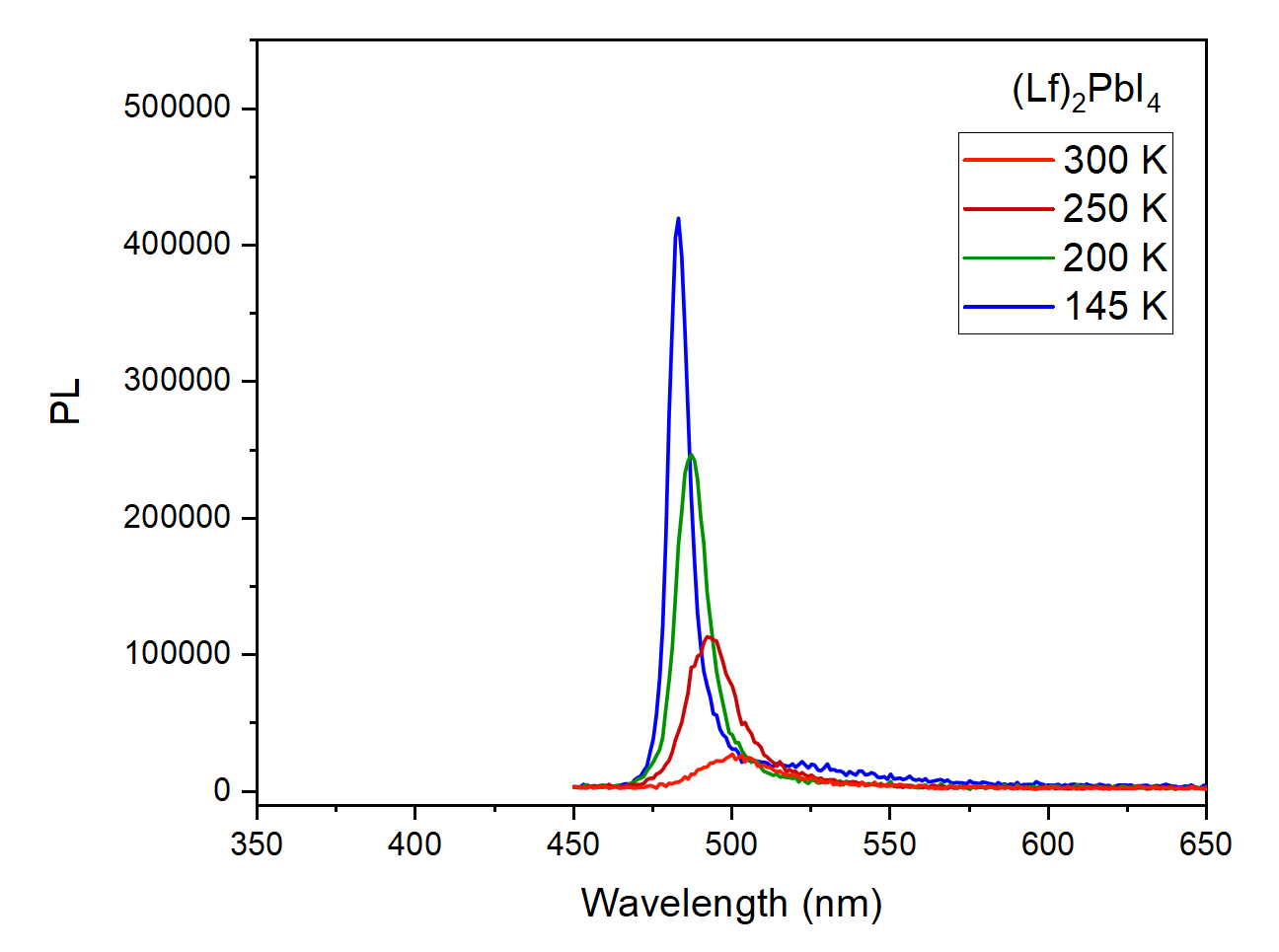


Figure S9. Not normalized PL spectra versus Temperature for the Lc and Lf thin films.

Crystallographic Information File (CIF) for the relaxed structure of (Lf)_2_PbI_4_

#======================================================================

# CRYSTAL DATA

#----------------------------------------------------------------------

data_VESTA_phase_1

_chemical_name_common                  ''

_cell_length_a                         9.0210

_cell_length_b                         8.6950

_cell_length_c                         53.500

_cell_angle_alpha                      90

_cell_angle_beta                       90

_cell_angle_gamma                      90

_space_group_IT_number                 61

loop_

_space_group_symop_operation_xyz

    '    x,    y,    z'

    '1/2-x,    y,1/2+z'

    '1/2+x,1/2-y,    z'

    '    x,1/2+y,1/2-z'

    '   -x,   -y,   -z'

    '1/2+x,   -y,1/2-z'

    '1/2-x,1/2+y,   -z'

    '   -x,1/2-y,1/2+z'

loop_

   _atom_site_label

   _atom_site_fract_x

   _atom_site_fract_y

   _atom_site_fract_z

Pb       0.000000000   0.000000000   0.000000000

I        0.673926312   0.167316844   0.001156412

I        0.031839852   0.997647096   0.062702277

N        0.079536804   0.602584954   0.052998337

C        0.970649626   0.575378087   0.074363230

C        0.938980387   0.403003793   0.076576302

C        0.855840050   0.355029422   0.100612120

C        0.961219561   0.192243618   0.244608736

C        0.941851490   0.392238077   0.124498055

C        0.897997648   0.292225550   0.147611207

C        0.035990799   0.218305575   0.218478972

C        0.923832744   0.239277416   0.196188429

C        0.993663740   0.312425813   0.171959210

F        0.889422009   0.322850430   0.252649875

F        0.860304329   0.073479911   0.243070826

F        0.125086932   0.348800061   0.220160629

F        0.066742119   0.154965908   0.262186687

F        0.126552805   0.092078865   0.213597646

F        0.131906129   0.244345846   0.167893705

F        0.865042606   0.095397540   0.190225891

F        0.807726984   0.333351264   0.204164190

F        0.015597531   0.468852978   0.176271444

F        0.749264601   0.323638028   0.153146121

F        0.907671129   0.137337059   0.140302092

F        0.096659494   0.373100748   0.120915995

F        0.921176659   0.549295235   0.130799620

H        0.041068931   0.558330884   0.036228808

H        0.871179694   0.642354871   0.069998749

H        0.872151426   0.365514295   0.060506889

H        0.043653591   0.339265400   0.075745434

H        0.838314484   0.230403754   0.099834679

H        0.746886907   0.410584194   0.102120536

H        0.023048068   0.624053855   0.090978958

H        0.183373473   0.554875855   0.056728659

H        0.093855714   0.720914196   0.050764747
